# Vitreous Hemorrhage Induced by a Persistent Hyaloid Artery

**DOI:** 10.1155/crop/6485755

**Published:** 2026-04-25

**Authors:** Henry Zou, Kimberly Drenser, Brooke Geddie

**Affiliations:** ^1^ Department of Undergraduate Medical Education, Michigan State University College of Human Medicine, Grand Rapids, Michigan, USA, msu.edu; ^2^ Associated Retinal Consultants, Grand Rapids, Michigan, USA, associatedretinalconsultants.com; ^3^ Department of Pediatric Ophthalmology, Corewell Health Helen DeVos Children’s Hospital, Grand Rapids, Michigan, USA

**Keywords:** persistent fetal vasculature (PFV), persistent hyaloid artery (PHA), retinopathy of prematurity (ROP), vitreous hemorrhage

## Abstract

**Background:**

The hyaloid artery is a branch of the ophthalmic artery and part of the fetal hyaloid vascular system; failure of these fetal vessels to regress can lead to a persistent hyaloid artery (PHA). PHA can induce complications, including amblyopia, cataracts, glaucoma, vitreous hemorrhage, and retinal detachment. We present a case of vitreous hemorrhage secondary to traction of the PHA in an infant treated for threshold retinopathy of prematurity (ROP).

**Case Presentation:**

A 660‐g female infant was born at 23 weeks of gestation and was found to have bilateral Stage 3 Zone 2 ROP with plus disease, prompting laser photocoagulation to the avascular retina. Six weeks after this treatment, the patient was found to have a vitreous hemorrhage requiring vitrectomy. The etiology of the hemorrhage was thought to be secondary to traction on the PHA after laser photocoagulation treatment. She developed subsequent anisometropic myopia and amblyopia, for which she continues care.

**Conclusions:**

Vitreous hemorrhage is a rare but severe complication of PHA. This unique case of traction on the PHA with subsequent vitreous hemorrhage after routine treatment of ROP highlights the importance of considering PHA in the management of ROP.

## 1. Introduction

The hyaloid artery (HA) is a branch of the ophthalmic artery that extends through the vitreous humor from the optic disk to surround the crystalline lens [1]. It is a component of the fetal hyaloid vascular system that supplies the inner retina and lens with oxygen and nutrients during gestation [1, 2]. Though the HA normally undergoes apoptosis following the formation of retinal vasculature during late embryologic development or shortly after birth, partial or complete failure of the hyaloid vascular system to regress results in a persistent hyaloid artery (PHA) [1]. PHA is found in 3% of full‐term infants but in 95% of premature infants and can mediate complications, including amblyopia, nystagmus, strabismus, cataracts, glaucoma, vitreous hemorrhage (VH), retinal detachment, and microphthalmia [1–4]. PHA is a subtype of persistent fetal vasculature (PFV), which is a broader term that describes the failure of fetal blood vessels such as the HA and the tunica vasculosa lentis to regress after birth [2]. Treatments vary depending on the type of PHA and its prognosis, ranging from observation to lensectomy, vitrectomy, residual stalk diathermy, and management of secondary complications such as amblyopia and glaucoma [2]. We present a case of VH induced by traction on the PHA in an infant treated for threshold retinopathy of prematurity (ROP).

## 2. Case Presentation

**Figure 1:**
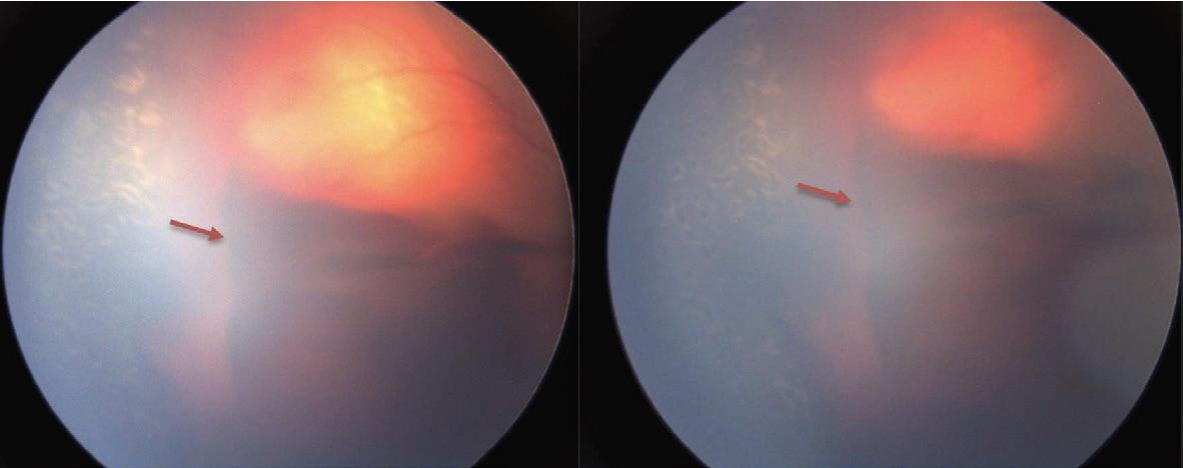
Right vitreous hemorrhage (red arrows) without retinal detachment.

A 660‐g premature female infant was born at 23 weeks of gestation. Her past medical history included pulmonary hypoplasia, patent ductus arteriosus, anemia of prematurity, feeding difficulties, dichorionic diamniotic twin gestation, and being large for gestational age. She received 225 days of supplemental oxygen therapy delivered via high‐ and low‐flow nasal cannula since birth. She was diagnosed with bilateral Stage 3 Zone 2 ROP with plus disease at 38.4 weeks of postmenstrual age (PMA) and underwent ROP laser photocoagulation of both eyes at 38.6 weeks of PMA. A red diode 810‐nm Oculight SLx laser (IRIDEX, Mountain View, California, United States) initially set to 150‐ms duration, 150‐mW power, and 300‐ms interval was used. Power was adjusted between 150 and 250 mW for adequate laser uptake, with 1200 applications to the right eye and 1176 applications to the left eye completed without complication. Postoperatively, both eyes responded well to treatment with regression of ROP. However, at 42.4 weeks of PMA, the ROP had resolved in the left eye but recurred as Stage 1 Zone 2 in the right eye. At 44.4 weeks of PMA, a large VH prohibited full direct visualization of the retina, and the patient was referred to a vitreoretinal specialist (Figure 1).

Vitrectomy was performed at 45.4 weeks of PMA. A perioperative exam confirmed a dense right VH extending inferiorly from the optic nerve with appropriate laser ablation to the avascular retina. Retinal detachment did not appear likely but could not be ruled out given the VH. A radial 12:00 conjunctival incision was made, and a peritomy was made at 9:00 and 3:00 at the limbus with sharp dissection. A 27‐gauge trocar was placed in the infratemporal quadrant 1 mm posterior to the limbus, and an infusion cannula was placed in this port. Sclerotomies were then made at 10:00 and 2:00 with a 27‐gauge MVR blade 1 mm posterior to the limbus using a 27‐gauge light pipe and vitrector. A core vitrectomy was performed. A patent PHA with traction on the retina was noted and considered the culprit for the VH. The PHA was bisected using a combination of extrusion and cutting, and the hyaloid was gently removed via lifting around the optic nerve out to the peripheral ridge until visual access was cleared. A 50% air–fluid exchange was performed, the infusion was removed, all sclerotomies were closed, the conjunctiva was repositioned and secured at 12:00, postoperative neomycin–polymyxin–dexamethasone ointment was applied, and an eye patch was placed.

The family was counseled to keep the patient in an upright position after surgery, tapered prednisolone acetate drops were prescribed for postoperative care, and weekly follow‐ups were scheduled. At 47.9 weeks of PMA, the right VH was observed to be clearing, and the ROP had regressed in the right eye. By 53.7 weeks of PMA, the hemorrhage had resolved, but residual heme was noted inferiorly, temporally, and nasally; moreover, there was vitreoretinal traction inferiorly but no signs of detachment. Though the patient was fully weaned off supplemental oxygen and prednisolone drops at this point, she developed deprivation amblyopia of the right eye and started patching therapy. By 66.3 weeks of PMA, the residual heme had fully resolved in the right eye. During subsequent close clinical follow‐up care, anisometropic amblyopia was diagnosed and treated with a glasses prescription and patching. Given difficulty with glasses compliance, contact lens fitting for the right eye was completed with successful treatment of anisometropia, with ongoing patching treatment for amblyopia.

## 3. Discussion

The etiology of PHA is not fully understood, though it is hypothesized that neuronal failure to upregulate vascular endothelial growth factor receptor 2 (VEGFR2) results in misdirected angiogenesis, thus hindering the apoptosis of the hyaloid vascular system [1, 3]. Normal hyaloid vascular regression requires the upregulation of VEGFR2 by neurons to limit angiogenesis and sequester excess VEGF, which then facilitates the physical separation of VEGF‐producing cells and the differentiation of lens epithelial cells [1, 3]. The overabundance of glial cells in the retrolental fibrovascular stalks has also been proposed as an etiologic mechanism according to a histologic review [2]. Finally, another hypothesis is that hyaloid vascular regression depends on proximal arterial vasoconstriction [5]. In oxygen‐treated murine models, the reversal of proximal arterial vasoconstriction by oxygen‐induced retinopathy induced the enlargement and tortuosity of distal hyaloid vasculature [5]. In our patient, it is possible that her extreme prematurity and long‐term supplemental oxygen therapy were both risk factors for PHA.

Though PHA‐associated VH is rare, it can be spontaneous or induced by rapid eye movement sleep, trauma, or posterior vitreous detachment [4]. VHs in infants can cause visual deprivation during the critical period of visual development, increasing the risk of deprivation amblyopia [4]. Moreover, the primary vitreous containing the PHA can proliferate and adhere to the retina, causing tractional retinal detachment and secondary macular abnormalities [2]. Our patient’s VH may have resulted from delayed‐onset traction on the PHA induced by retinal contraction after ROP laser photocoagulation.

PFV is categorized into anterior, posterior, and combined types; anterior PFV involves structures in front of the lens, posterior PFV involves the vitreous and retina, and combined PFV involves fibrovascular tissue extending from the retina and vitreous to the lens [6]. Combined PFV is further subtyped into I, Y, inverted Y, and X, each describing the morphology of the vascular stalk as visualized by B‐mode ultrasound and color Doppler imaging [7]. Subtype I refers to a singular, unbranched vascular stalk that extends from the optic nerve to the posterior lens capsule [7]. Subtype Y refers to a vascular stalk that extends from the optic nerve as a single vessel but then divides into two branches as it travels anteriorly toward the lens [7]. Subtype inverted Y refers to when the vascular stalk has two separate origins in different parts of the retina or optic nerve head but fuses into a single stalk before reaching the lens [7]. Finally, subtype X refers to a multibranched vascular stalk that features the intersection of multiple vascular remnants in the midvitreous, thereby increasing the risk of vitreoretinal traction [7]. Our patient’s PHA was best classified as subtype X given its multibranched vascular stalk that caused vitreoretinal traction, ultimately inducing VH.

Early vitrectomy for PHA‐induced VH is vital for reducing the risk of secondary complications, but surgical approaches vary depending on whether the PHA is in the anterior segment, posterior segment, or both (combined) [2]. The anterior approach is preferred for anterior PHA or combined PHA type Y or X to avoid iatrogenic damage to the ora serrata or peripheral retina [2]. However, the pars plicata approach is preferred for posterior PHA or combined PHA type I or inverted Y, as the pars plana is too narrow in children [2]. Furthermore, the pars plicata approach is necessary in premature infants due to the underdevelopment of the pars plana, resulting in the lens equator extending more posteriorly [8]. This makes the traditional pars plana approach unsafe as it risks iatrogenic lens damage during instrument insertion [8]. In the pars plicata approach, sclerotomies used to access the vitreous cavity are typically placed 0.5–1.0 mm posterior to the limbus to avoid lens damage [8]. After entry sites are precisely localized, the MVR blade is initially directed perpendicular to the globe and then angled toward the eyeball center after passing the lens equator [8].

Rarely, persistent PHA hemorrhages may require residual stalk amputation or endodiathermy [2]. Nonetheless, intensive long‐term amblyopia management is common even after successful surgical intervention [2]. Although our patient experienced complete VH resolution after vitrectomy and did not have retinal detachment, she did, as anticipated, develop deprivation amblyopia in the affected eye.

## 4. Conclusions

PHA is common in premature infants, and VH is a rare but severe secondary complication that can mediate visual deprivation during critical periods of visual development and tractional retinal detachment if untreated. We present the case of PHA‐induced VH in an extremely premature infant with ROP receiving supplemental oxygen therapy and undergoing laser photocoagulation treatment for threshold ROP. This case highlights the importance of considering possible PHA‐related complications during the necessary treatment of ROP.

## Funding

No funding was received for this manuscript.

## Ethics Statement

The authors have nothing to report.

## Consent

All the patients allowed the processing of their personal data, and informed consent was obtained from all individual participants included in the study.

## Conflicts of Interest

The authors declare no conflicts of interest.

## Data Availability

The data that support the findings of this study are available on request from the corresponding author. The data are not publicly available due to privacy or ethical restrictions.
